# Case Report: SARS-CoV-2 Mother-to-Child Transmission and Fetal Death Associated With Severe Placental Thromboembolism

**DOI:** 10.3389/fmed.2021.677001

**Published:** 2021-08-16

**Authors:** Penélope Saldanha Marinho, Antonio José Ledo Alves da Cunha, Leila Chimelli, Elyzabeth Avvad-Portari, Felipe da Matta Andreiuolo, Patrícia Soares de Oliveira-Szejnfeld, Mayara Abud Mendes, Ismael Carlos Gomes, Letícia Rocha Q. Souza, Marilia Zaluar Guimarães, Suzan Menasce Goldman, Mariana Barros Genuíno de Oliveira, Stevens Rehen, Joffre Amim, Fernanda Tovar-Moll, Arnaldo Prata-Barbosa

**Affiliations:** ^1^Maternity School, Federal University of Rio de Janeiro, Rio de Janeiro, Brazil; ^2^Department of Pediatrics, D'Or Institute for Research and Education, Rio de Janeiro, Brazil; ^3^Laboratory of Neuropathology, Rio de Janeiro State Brain Institute, Rio de Janeiro, Brazil; ^4^Department of Pathological Anatomy, Fernandes Figueira Institute, Fiocruz, Rio de Janeiro, Brazil; ^5^School of Medicine, State University of Rio de Janeiro, Rio de Janeiro, Brazil; ^6^Diagnostic Imaging Department, Escola Paulista de Medicina, School of Medicine, Universidade Federal de São Paulo, São Paulo, Brazil; ^7^Diagnostic Imaging Department, D'Or Institute for Research and Education, Rio de Janeiro, Brazil; ^8^Stem Cell Laboratory, D'Or Institute for Research and Education, Rio de Janeiro, Brazil; ^9^Genetics Department, Institute of Biology, Federal University of Rio de Janeiro, Rio de Janeiro, Brazil; ^10^Anatomic Pathology Service, Jesus Municipal Hospital, Rio de Janeiro, Brazil; ^11^Department of Phamacology, Institute of Biomedical Sciences, Federal University of Rio de Janeiro, Rio de Janeiro, Brazil

**Keywords:** SARS-CoV-2, COVID-19, pregnancy, fetal death, placental thromboembolism, mother-to-child transmission

## Abstract

SARS-CoV-2 infection during pregnancy is not usually associated with significant adverse effects. However, in this study, we report a fetal death associated with mild COVID-19 in a 34-week-pregnant woman. The virus was detected in the placenta and in an unprecedented way in several fetal tissues. Placental abnormalities (MRI and anatomopathological study) were consistent with intense vascular malperfusion, probably the cause of fetal death. Lung histopathology also showed signs of inflammation, which could have been a contributory factor. Monitoring inflammatory response and coagulation in high-risk pregnant women with COVID-19 may prevent unfavorable outcomes, as shown in this case.

## Introduction

Initial studies of COVID-19 during pregnancy pointed to the non-transmission or unusual transmission of SARS-CoV-2 to the fetus (1, 2). However, other publications have demonstrated the probable mother-to-child transmission due to the detection of IgM antibodies (3) or positivity of RT-PCR in newborns a few hours after birth (4–6). One study reported five cases of fetal deaths and demonstrated positive viral RT-PCR in the amniotic fluid in one case and in the placenta in two cases (7). Despite this indirect evidence of SARS-CoV-2 vertical transmission, no study has yet comprehensively demonstrated the presence of the virus in fetal tissues. In this case report, we describe a documented mother-to-child transmission of SARS-CoV-2, demonstrating the widespread detection of the virus in the placenta, umbilical cord, and several fetal tissues. We also found severe placental thromboembolic involvement through studies of fetal–placental magnetic resonance imaging (MRI) and histopathology of the placental, which is the probable cause of fetal death. Subsequent screening for thrombophilia did not indicate significant risk factors in this patient, suggesting that placental thromboembolic complications were mainly determined by infection with the new coronavirus (COVID-19).

## Case Description

A 33-year-old black woman at 34 weeks and 4 days of gestation was admitted on October 15, 2020, with a diagnosis of fetal death 14 days after a positive RT-PCR for SARS-CoV-2. The patient (gravida 2, para 1) was 1.80 m tall, weighed 87.7 kg (body mass index (BMI) 27.1 kg/m^2^), and had a history of previous bariatric surgery (Roux-en-Y gastric bypass in 2012). She started her prenatal care in the 9th week of pregnancy. She regularly used omeprazole to treat gastritis, multivitamins, ferrous sulfate, folic acid, and vitamin D. She denied smoking, alcoholism, or the use of illicit drugs. The cardiovascular exam showed a regular rhythm, 79 bpm, without cardiac murmur and blood pressure level 120 × 70 mmHg. The respiratory clinical examination and laboratory tests were normal, including serology for HIV, toxoplasmosis, and syphilis. The vaccination history showed a complete schedule for tetanus, hepatitis B, and influenza. Morphological ultrasound at 21 weeks and 1 day of gestation, performed on July 13, 2020, was normal, as well as Doppler ultrasonography performed at 28 weeks and 1 day.

Two weeks before admission (October 1, 2020), she complained of flu-like symptoms at a routine prenatal visit, which started on September 25, 2020, when she had myalgia, runny nose, headache, and fatigue. She denied fever and contact with a suspected or confirmed case of COVID-19. The physical examination showed a stable general condition, respiratory rate 25/min, without dyspnea, oxygen saturation of 99% on room air, heart rate 112 bpm, and without fever or other significant findings. Ultrasonography estimated fetal weight at 2,156 g (64.6% percentile), cephalic presentation, 146 bpm fetal heart rate, normal amniotic fluid volume, and elevated posterior grade II placenta. Doppler evaluation was normal, with umbilical artery pulsatility index (PI) 0.60, middle cerebral artery PI 1.66, and cerebroplacental ratio 2.77. The rapid serological tests for SARS-CoV-2 (IgM and IgG, colloidal gold immunochromatography test, Beijing LEPU-Medical Technology, Beijing, China) were negative, but the qualitative RT-PCR on naso- and oropharyngeal swabs [Bio Manguinhos Molecular Kit SARS-CoV-2 [E/RP], Charité Berlin Protocol (8)] was positive (result released on October 3, 2020). The patient was instructed to remain in isolation at home for 14 days and return to the emergency room in case of signs or symptoms of worsening.

On October 15, 2020, she returned to prenatal care without symptoms of COVID-19 for the previous 8 days. She reported that on October 4, she had a temperature of 37.2°C and maintained flu-like symptoms until October 8, although afebrile. On physical examination, fetal heartbeats were not detected, and the diagnosis of fetal death was confirmed by ultrasound examination, which estimated gestational age at 34 weeks and 4 days. On that occasion, a new rapid serological test (same manufacturer) was positive for IgM and IgG anti-SARS-CoV-2. The induction of delivery was scheduled for the following day. The patient was then submitted to an MRI for better evaluation of fetal and placental morphology and collected new laboratory tests, including serology for SARS-CoV-2 and TORCH (toxoplasmosis, rubella, cytomegalovirus (CMV), herpes simples, parvovirus, Zika (ZIKV), and chikungunya viruses), in addition to blood biochemistry and dosage of inflammatory biomarkers (Table 1). On October 16, she underwent an elective cesarean delivery because she declined to induce labor. The stillbirth weighed 2,145 g, and the amniotic bag was ruptured during the operation, giving rise to a brownish-looking liquid.

**Table 1:** Main laboratory tests at the time of delivery and thrombophilia screening 4 months later.

	**Results**	**Reference range**
Blood count		
Red cells (millions/mm^3^)	4.27	4.30–5.70
Hemoglobin (g/dl)	12.4	12.0–17.5
Hematocrit (%)	39.4	35.0–50.0
Total leukocytes (per mm^3^)	17,500	3,500–10,500
Neutrophils (%)	83	50–70
Lymphocytes (%)	13	25–40
Monocytes (5)	4	8–13
Platelets (per mm^3^)	409,000	150,000–450,000
Coagulation		
D-dimer (ng/ml)	17,216	Up to 500
Fibrinogen (mg/dl)	353	200–393
aPTT (s)	35.8	29.0
PT (s)	9.8	12.3
INR	1.0	0.9–1.2
Inflammatory biomarkers		
CRP (mg/dl)	0.90	Up to 1
ESR (mm/h)	25	Up to 10
Ferritin (ng/ml)	323.3	10–291
Interleukin 6 (pg/ml)	71.7	<7
Interleukin 1-beta (pg/ml)	10.0	<5
Interleukin 10 (pg/ml)	12.2	<9.1
TNF (pg/ml)	10.1	<8.1
VEGF (pg/ml)	<31	31–86
TGF-B (ng/ml)	20.4	1.4–8.2
Serology		
Anti-SARS-CoV-2 IgG (S/C)^a^	20.1	<1.0
Anti-SARS-CoV-2 Total (S/C)^a^	115.0	<1.0
Anti-toxoplasmosis IgM (index)/IgG (UI/ml)^b^	<0.5/ <0.25	<0.5/ <1.6
Anti-rubella IgM (index)/IgG (UI/ml)^c^	<0.8/101.3	<0.8/ <10.0
Anti-CMV IgM/IgG (U/ml)^c^	<0.7/>500.0	<0.7/ <0.5
Anti-herpes simplex IgM/IgG (index)^c^	0.5/30.0	<0.9/ <0.9
Anti-parvovirus B19 IgM/IgG (U/ml)^d^	<5.0/ <2.0	<10.0/ <3.0
Anti-ZIKV IgM/IgG	Neg/Pos	Neg/Neg
Anti-CHIKV IgM/IgG	Neg/Neg	Neg/Neg
Amniotic fluid		
Leukocytes (per mm^3^)	3,750	<2
Polymorphonuclear (%)	6	-
Mononuclear (%)	94	-
Red blood cells (per mm^3^)	102,500	<1,000
Normal (%)	95	-
Crenated (%)	5	-
LDH (U/L)	6,573	<250
Thrombophilia screening (four months after delivery)		
Leiden Factor V G1691A mutation	Heterozygous	Not present
MTFHR C677T and A1298C mutations	Not present	Not present
FII G20210A mutation	Not present	Not present
CBS 844ins68 polymorphism	Not present	Not present
PAI-1 4G/5G polymorphism	5G/5G Homozygous	5G/5G Homozygous
Lupus anticoagulant antibody	0.8	≤ 1.2
Protein C (%)	74	70–140
Protein S (%)	63	59–118
Homocysteine (μmol/L)	15.0	<15
IgA anti-cardiolipin antibodies (APL U/ml)	1.6	<14
IgM anti-cardiolipin antibodies (MPL U/ml)	0.9	<10
IgA anti-β2glycoprotein I antibodies (U/ml)	6.2	<7
Antithrombin III activity (%)	99.9	79.4–112
PAI-1 gene mutation	Negative (5G/5G)	(5G/5G)

*aPTT, activated partial thromboplastin time; PT, prothrombin time, INR, international normalized ratio; CRP, C-reactive protein; ESR, erythrocyte sedimentation rate; TNF, tumor necrosis factor; VEGF, vascular endothelial growth factor; TGF-B, transforming growth factor beta; SARS-CoV-2, severe acute respiratory syndrome caused by the coronavirus type 2; CMV, cytomegalovirus; ZIKV, Zika virus; CHIKV, chikungunya virus; LDH, lactic dehydrogenase; PAI-1, plasminogen activator inhibitor 1; MTFHR, methylene-tetrahydrofolate reductase; CBS, cystathionine β-synthase*.

a *Immunochemoluminescence*.

b *CMIA, chemiluminescent microparticle immunoassay*.

c *CLIA, chemiluminescent immunoassay*.

d *ELISA, enzyme-linked immunosorbent assay*.

Four months later, a complete screening for thrombophilia was performed. The results showed a single alteration of a heterozygous pattern for Leiden Factor V. A timeline of the case evolution is presented in Supplementary Figure 1.

### Patient Perspective

The patient felt the loss of her daughter very much but felt that studying her case could help other families. She recently received the first dose of the anti-COVID-19 vaccine (AstraZeneca), and a few weeks later, she learned that she was pregnant again. Now, she is 7 weeks pregnant (June 2021) and very hopeful for her new opportunity.

### Fetal–Placental Magnetic Resonance Imaging

**Figure 1:**
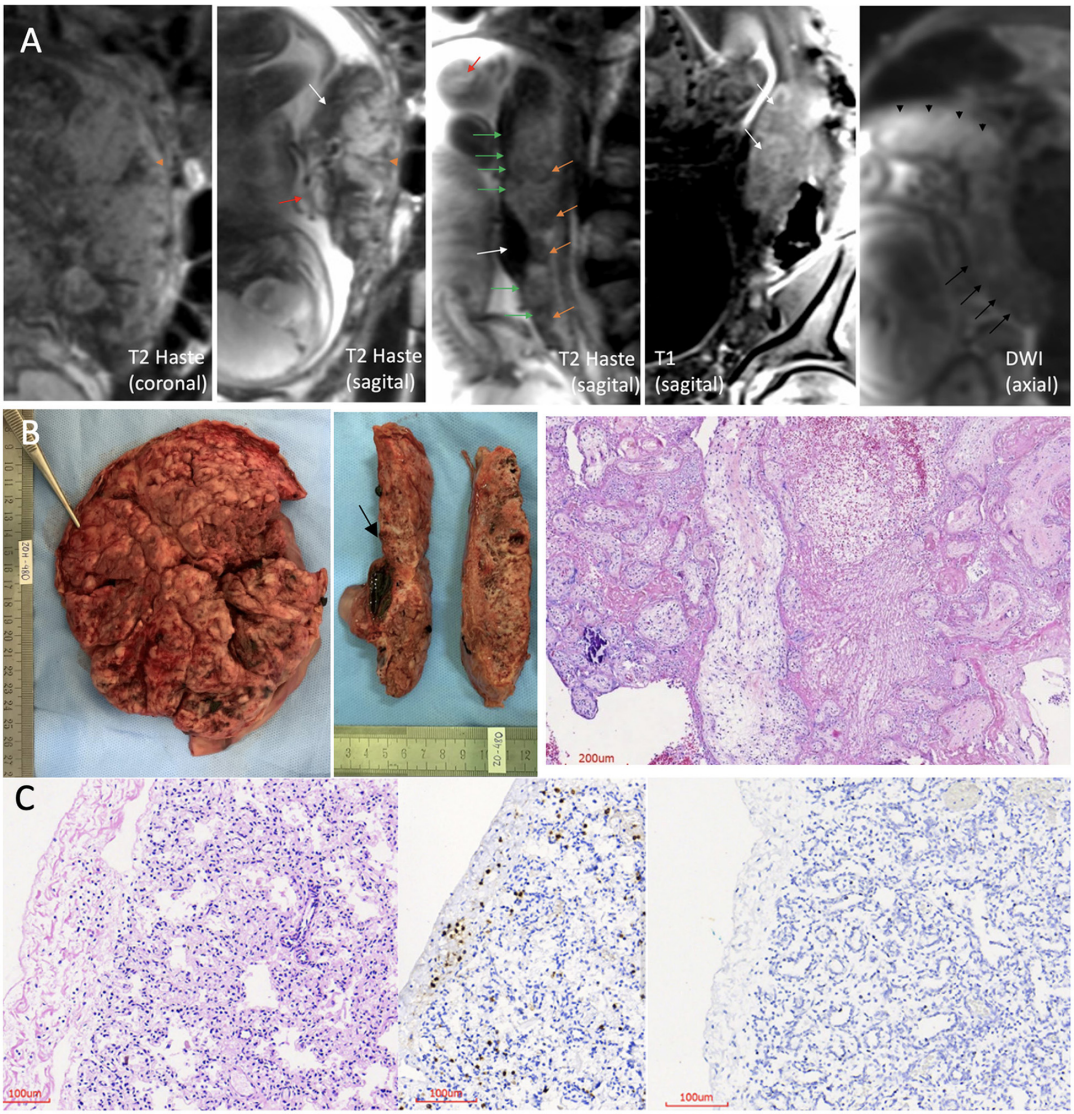
Fetal–placental magnetic resonance imaging (MRI); pathology of the placenta and lung histopathology. **(A)** 3.0 Tesla MRI of placenta and fetus, using two 18-channel abdominal coil, showing the umbilical cord with no evident flow, no evident signal void (red arrow), and heterogeneous placenta, with ill-defined cotyledons, with contours having fewer lobulations than usual and wedge-shape or round-shape hypointense signal areas in T2 Haste sequence (orange arrowhead and orange arrows, respectively). The placental image showed insertion in the posterior wall, with a “cake” aspect, and the uterine insertion base is smaller than usual, which gave it a “shrinkage” aspect (coronal and sagittal T2 Haste images). Altered habitual T1 and T2 placental signals with hyposignal plate in T2 along its entire length of the fetal face, appearance of a line drawn in pencil (green arrows), presence of large and dilated vessels with massive thrombosis characterized by the unusual hyperintense signal in T2, and also blood lakes between the placental tissue (areas of hypointense signal in T2 and hyperintense signal in T1, predominating in the periphery) (white arrows). Diffusion-weighted image (DWI) sequence shows higher restriction than the usual pattern, and this is more evident in the right posterolateral portion of the placenta (black arrowheads), where vascular dilation and thrombosis are more pronounced. There are also areas of necrosis characterized by the facilitation mechanism more evident in the left placental portion (black arrows). **(B)** Placenta pathology. Left: maternal surface is wrinkled, compact, and yellow-tan with patchy hemorrhages. Middle: cut surfaces with extensive and diffuse fibrin deposition and recent infarcts. At the level of the umbilical cord insertion, there is a large subchorionic thrombus (arrow). Right: histological section with villitis, intervillositis, and massive fibrin deposition with intervillous thrombosis. **(C)** Histological sections of the lung. Left: thick alveolar septa with aggregates and scattered lymphocytes in pleura (hematoxylin and eosin). Middle: immunohistochemistry showing CD3+ T lymphocytes in the pleura and alveolar septa. Right: immunohistochemistry showing no lymphocytes in a normal control lung (patient without COVID-19).

An anatomical and advanced MRI protocol with multiple sequences was performed in a 3.0 Tesla MRI scanner (Magnetom Prisma, Siemens Healthcare, Erlangen, Germany), using two 18-channel abdominal coils for detailed analyses of the placenta and fetal organs in the three orthogonal planes. The complete protocol used is described in the Supplementary Material. The main findings are presented in more detail in Figure 1A and Supplementary Figure 2 and consisted mainly of a substantial placental impairment (appearance of a collapsed placenta with no viability). The placenta had a posterior uterine wall insertion, ill-defined cotyledons, fewer lobulations than usual, and large and dilated vessels with massive thrombosis. The fetus presented accumulation of fluid in the pericardium, and pleural and peritoneal space; alterations in the heart, liver, and spleen images; and signs of diffuse cerebral edema.

### Anatomopathological Examination of the Placenta

The placenta weighted 470 g (80th percentile for gestational age) and had a paramarginally inserted three-vessel umbilical cord with a reddish color, and the chorionic plate vessels were highly dilated and congested and had magistral distribution; the fetal surface was reddish, and the membranes were hypotransparent (Figure 1B, left and middle). The maternal surface was compact, wrinkled, and yellow-tan with patchy hemorrhages, resembling maternal floor infarction and poorly delimited and frayed lobes. Cut sections demonstrated extensive whitish irregular deposits, areas of intervillous and subchorionic thrombi, and recent infarcts. Histologically, it showed massive diffuse inflammatory alterations in regions with a predominance of acute and chronic intervillositis (Supplementary Figures 3F,G) and intervillous neutrophilic microabscesses associated with chronic and proliferative villitis and acute deciduitis (Figure 1B, right; and Supplementary Figure 3F). There were also signs of severe maternal vascular malperfusion represented by extensive and diffuse intervillous/perivillous fibrin deposition with areas of intervillous laminated thrombi and a large subchorionic recent thrombus at the umbilical insertion (Supplementary Figure 3C). Areas of recent infarct characterized by collapsed and necrotic villi with partial or total loss of trophoblast layer (Supplementary Figure 3H) and hypovascular or avascular stroma were observed. The decidua showed focal laminar necrosis and some vessels with fibrinoid necrosis and thrombi. Signs of acute fetal vascular malperfusion, including ectasia and recent thrombi, were noted in large vessels from the chorionic plate and stem villous vessels (Supplementary Figure 3C). There were also stem villous vessels with subintimal fibrin deposition and occlusive thrombus with recanalization (Supplementary Figure 3D) and few villi with stromal-vascular karyorrhexis (Supplementary Figure 3E). Occasional groups of preserved chorionic villi showed delayed villous maturation.

### Fetus Autopsy Findings

The organs were fixed in 10% buffered formalin, and fragments of all sampled tissues were processed for paraffin embedding and were stained with hematoxylin and eosin. The skin was detached from the surface of the body due to postmortem autolysis compatible with the estimated time of fetal death (>48 h) (Supplementary Figure 4). All organs of the thoracic and abdominal cavities were congested and soft due to postmortem autolysis (Supplementary Figure 5). At removal, the brain weighed 280 g. The gyri were wide, congested, and extremely soft due to autolysis (Supplementary Figure 6). Histologically, the brain and spinal cord showed no signs of inflammation or necrosis. The meninges and choroid plexus were immunostained for T lymphocytes, which was negative. The olfactory bulb had occasional T lymphocytes. The pituitary gland presented small foci of calcification. The vagus nerve and dorsal root ganglia were normal, as were the muscles (psoas, diaphragm, and abdominal). Histology of the lungs showed mild thickening of the pleura and alveolar septa, with mild infiltration of lymphocytes (Figure 1C, left) without signs of fibrosis as seen with Gomori's staining. Immunohistochemistry for leukocyte common antigen (LCA) showed small aggregates of lymphocytes in pleura and pulmonary septa. They were CD3 T lymphocytes (Figure 1C, middle) and can be compared with a negative control lung of a patient without COVID-19 (Figure 1C, right). In the kidneys, occasional interstitial lymphocytes immunostained with LCA were seen. Most of the other organs showed signs of maceration, particularly the liver, adrenal gland, spleen, pancreas, and intestines.

### Detection of SARS-CoV-2 in the Placenta and Fetus

Freshly collected samples from the fetal and maternal faces of the placenta, umbilical cord, heart, lungs, liver, kidney, intestines, cerebellum, olfactory bulb, and brain cortex were properly stored in RNA stabilization solution and later processed for viral RNA extraction and identification by RT-PCR. The complete experimental protocol, positive and negative controls, and standards for result interpretation are described in the Supplementary Material. Results of the RT-PCR assay were considered positive if either N1 or N2 SARS-CoV-2 nucleocapsid fragments had a cycle threshold of <40 (9). The standard curve and data analysis were prepared as described previously (10). The mean and standard deviations of absolute quantification (number of copies/reactions) of N1 and N2, and the mean and standard deviations of crossing points (Cp) values of Hs_RPP30 (*Homo sapiens* Ribonuclease P protein subunit p30) were calculated from data obtained in all analyzed distinct fragments from the same postmortem tissue.

**Figure 2:**
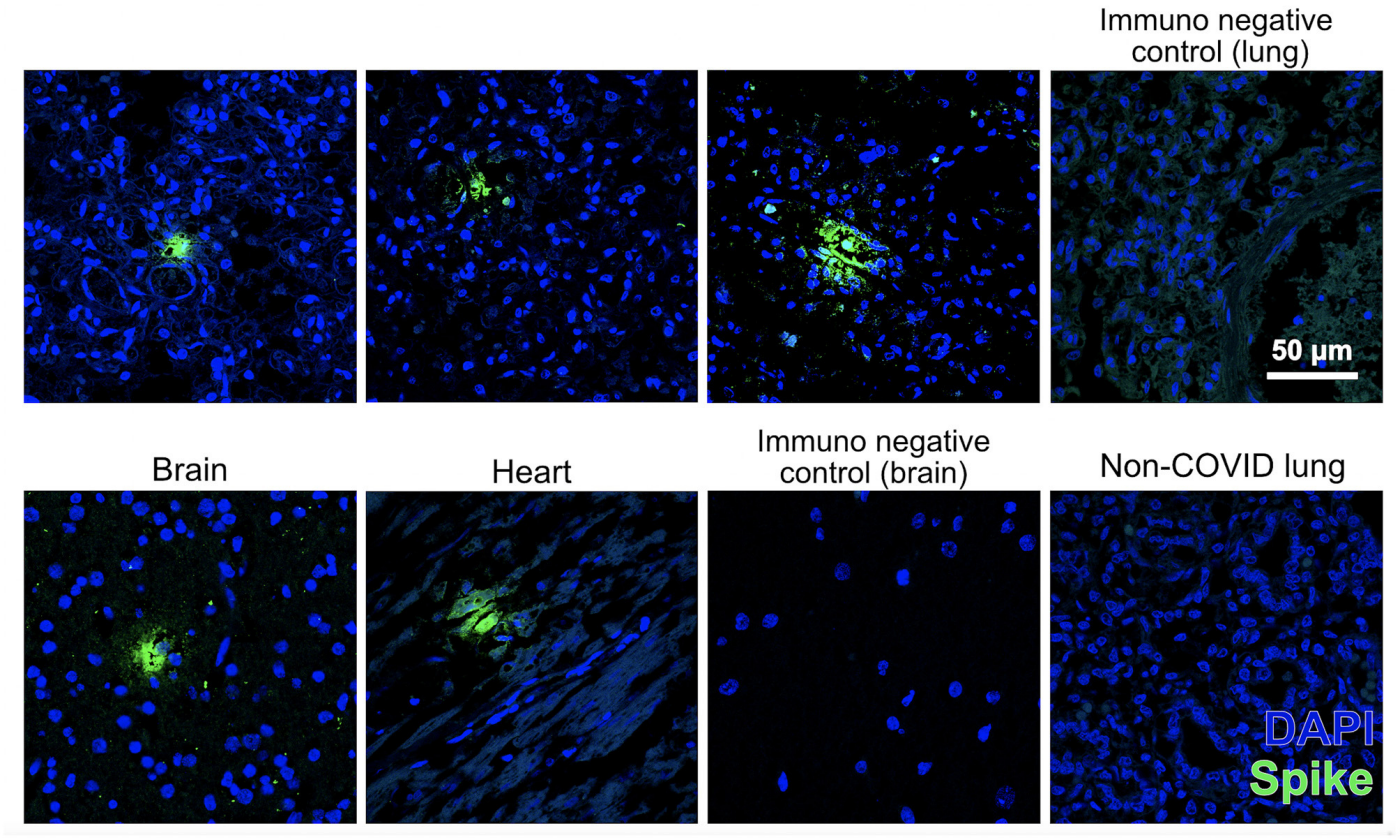
Fetal tissues immunostained for SARS-CoV-2 spike protein (SP) (green) counterstained with nuclear marker DAPI (blue). Images were acquired with a confocal microscope Leica TCS SP8 using a × 63 objective lens. Scale bar for all pictures is 50 μm. Top pictures: pulmonary immunodetection of SP (the three on the left) and negative control of immunofluorescence without primary antibody (right) in the present case. Lower images: immunodetection of SP in the brain and heart of this case (the two on the left), negative control immunofluorescence without primary antibody in a section of the brain, and lack of immunodetection of SP in a non-COVID fetal lung (right).

SARS-CoV-2 was detected in high amounts in the placenta. These amounts were also at least an order of magnitude higher than the average for nasopharyngeal swabs (positive control). Additionally, the new coronavirus was detected by qRT-PCR in the umbilical cord and the following fetal tissues: the salivary gland, trachea, olfactory bulb, lungs, liver, and kidney, but not in the heart or brain (Table 2). To confirm the presence of the virus in fetal tissues, sections of the lung, heart, and brain were immunostained against spike protein. As a result, positive immunolabeling was identified in the fetus's lung, brain, and heart and was absent in the immunofluorescence negative lung control of a non-COVID case (Figure 2).

**Table 2:** RT-qPCR detection of SARS-CoV-2 in the fetal tissues.

**Specimen type and number of fragments (n)**	**2019-nCoV N1 assay (mean copies ± SD)**	**2019-nCoV N2 assay (mean copies ± SD)**	**Hs_RPP30 (Ct mean ± SD)**	**Result interpretation (2019-nCoV detected/total tested)**
Brain (1)	ND	ND	31.6	Not detected (1/1)
Olfactory bulb (1)	ND	15	16.6	Detected (1/1)
Salivary gland (3)	21 ± 5.1	<10	21 ± 0.02	Detected (3/3)
Trachea (3)	1,050	ND	16.2 ± 0.3	Detected (1/3)
Lung (1)	212	ND	15.0	Detected (1/1)
Heart (1)	ND	ND	15.0	Not detected (1/1)
Liver (1)	196	47	18.7	Detected (1/1)
Kidney (1)	ND	<10	21.4	Detected (1/1)
Placenta, maternal face (3)	3.4 × 10^8^ ± 4.4 × 10^7^	2.6 × 10^8^ ±2.8 × 10^7^	17.7 ± 0.05	Detected (3/3)
Placenta, fetal face (2)	5 × 10^9^ ± 6.2 × 10^7^	3.9 × 10^9^ ±1.2 × 10^8^	14.8 ± 0.02	Detected (2/2)
Umbilical cord (2)	126	11	23.5	Detected (1/1)
**Controls**				
No template control	ND	ND	ND	Not detected (2/2)
Human specimen control (negative control)	ND^a^	ND^a^	23.2^a^	Not detected (1/1)
	ND^b^	ND^b^	17.3 ± 0.1^b^	Not detected (3/3)
	ND^c^	ND^c^	31.3 ± 0.7^c^	Not detected (2/2)
Human specimen control (positive control)^c^	21,945 ± 15,685	28,626 ± 17,309	30.6 ± 0.7	Detected (2/2)
2019-nCoV_N (positive control)^c^	3,858 ± 394	8,195 ± 1,612	ND	Detected (2/2)
Hs_RPP30 (positive control)	ND	ND	24.7 ± 0.06	Not detected (2/2)

*Ct values and interpretation*.

*RT-qPCR, quantitative reverse transcription polymerase chain reaction; SARS-CoV-2, severe acute respiratory syndrome coronavirus 2; Ct, cycle threshold; 2019-nCoV, CDC 2019 novel coronavirus real-time RT-PCR diagnostic panel; N1 and N2, SARS-CoV-2 nucleocapsid fragments; RP, internal control (internal RNase P target); Hs_RPP30, Homo sapiens Ribonuclease P protein subunit p30; ND, not determined*.

a *Pediatric postmortem kidney tissue*.

b *Human neural stem cells*.

c *Adult nasopharyngeal swab*.

## Discussion

In this case report, we describe a fetal death associated with a recent maternal infection by SARS-CoV-2. Viral RNA was detected in abundance in the placenta and umbilical cord and several fetal tissues. The severe placental abnormalities demonstrated by both the MRI and the anatomopathological study are consistent with intense acute fetal vascular malperfusion, probably the cause of fetal death, triggered by a severe maternal systemic inflammatory response to SARS-CoV-2 infection.

The impact of SARS-CoV-2 infection during pregnancy remains not completely clear, but some studies suggest that the infection may not lead to unfavorable maternal and neonatal outcomes (11, 12). Other papers support the hypothesis that *in utero* SARS-CoV-2 mother-to-child transmission, although rare, is possible, as SARS-CoV-2 genome was already detected in amniotic fluid and first-trimester placenta (fetal membranes) in association with hydrops fetalis (13), and in umbilical cord blood, at-term placentas, and neonatal nasopharyngeal swabs after birth (14). In addition, the intense inflammatory response at both systemic and placental levels can be extended to the fetus (15).

In the present case, like most pregnant women infected with SARS-CoV-2 (16, 17), our patient had mild symptoms during COVID-19, and the fetal evaluation was normal at the time of diagnosis. But on the other hand, she had a history of previous bariatric surgery and was still overweight, which placed her in the high-risk group for complications (18). In addition, some studies suggest that when the disease occurs in late pregnancy, it is associated with an increased risk of adverse outcomes, including premature birth (19–21). At the time of the fetal death diagnosis, despite the patient being clinically stable, laboratory tests revealed a pro-inflammatory and pro-coagulant syndrome, characterized mainly by an increase in interleukins, ferritin, and D-dimer, the latter at an exceptionally very high level. It is known that COVID-19 may predispose to a thrombotic condition (venous and arterial) due to inflammation, platelet activation, endothelial dysfunction, and blood stasis (22–24). This hypercoagulability state might intuitively play an essential role in pregnancy due to its inherent pro-thrombotic condition and might represent an additional risk for perinatal complications. Although D-dimer levels increase progressively in pregnancy and a level of 2,000 ng/ml can still be within the normal range for pregnant women, our patient had levels 30 times higher than the normal upper limit, which may have contributed to the impairment of placental perfusion and the severe abnormalities observed in the placenta. Although it cannot be completely ruled out, it seems unlikely that such findings were due to previous thrombophilia, since screening tests performed 4 months after pregnancy showed only heterozygosity for Leiden Factor V, which has not been associated with adverse pregnancy outcomes (25), representing a risk of <10% for thromboembolic disease (26). The possibility of other concomitant infectious diseases as determinants of the fetal outcome seems unlikely, given the negative results for the most common infectious conditions in pregnancy. The detection of positive IgG for CMV and ZIKV probably refers to a previous state, given the negative IgM result for these viruses, the local prevalence for CMV, and the recent ZIKV epidemic.

MRI was performed in this case as part of an ongoing trial to study COVID-19 fetal and prenatal manifestations and has shown that it can be a valuable test for better assessing the placenta of pregnant women with high-risk factors and COVID-19. The findings in this case showed good correspondence with the anatomopathology. Image and pathology findings in vascularization of the placenta suggested a massive reduction in fetal blood circulation, which alone is sufficient to explain fetal death (7, 27, 28). Fluid accumulation in the fetus is common in some viral infections and has already been reportedly associated with COVID-19 in pregnancy (29).

Angiotensin-converting enzyme 2 (ACE2) is the receptor of SARS-CoV-2 and plays an essential role in human infection and transmission. Some papers demonstrated *in situ* expression of ACE2 in the placenta and fetal tissues, such as the kidneys, liver, and lungs, reflecting the possibility of mother-to-child transmission and infection of fetal organs (30–32). Indeed, SARS-CoV-2 was detected in all sample extractions from the placenta, with massive numbers of copies. Viral RNA was also detected by qRT-PCR in the umbilical cord, kidneys, liver, salivary gland, lungs, trachea, and olfactory bulb, but not in the heart. Therefore, the possibility of direct harmful viral effects on fetal organs cannot be excluded as a complementary cause for death. We detected both the N1 and N2 SARS-CoV-2 targets in the liver and salivary gland but not in the trachea, lungs, or kidney. The reasons for these differences in analytical sensitivity between CDC-designed primer-probe sets N1 and N2 can be many (33, 34). In the present case, based on the analysis of Ct values, discrepant results between specimen types appeared to occur in samples with low viral load and close to the detection limit and/or with viral RNA degradation.

An essential finding of this study was identifying the spike protein of SARS-CoV-2 through the immunofluorescence technique in several tissues, such as the lungs, brain, and heart. The immunostaining corroborated the qRT-PCR findings that there were indeed viruses in fetal tissues. The fact that we found immunostained loci in the heart and the brain, whereas nothing was detected in these tissues by qRT-PCR, was unexpected but not entirely unplausible. As we argued above, RNA is very sensitive to degradation, and therefore, proteins can outlast this macromolecule in the context of extensive autolysis. Moreover, if the virus only infected a few cells or regions of the heart or brain, whatever little RNA survived after several days of infection might get diluted in an organ homogenate. In contrast, with histology, we can detect discrete regions where viral proteins endured longer.

Our findings of important inflammatory placental lesions (acute and chronic intervillositis, villositis, and acute deciduitis), poor maternal vascular perfusion (intense intervillous fibrin deposition with diffuse collapse of the intervillous space, resulting in necrosis of the chorionic villi—acute infarcts—and intervillous laminated thrombi), and extensive syncytiotrophoblastic damage (lysis) have already been described in similar cases (13, 35, 36) of SARS-CoV-2 infection in pregnant women. We present a complete panel of placental histopathology in the Supplementary Material.

As limitations of our study, we highlight the following: a) it was not possible to detect viral RNA or human RNA control (RP) in the brain and cerebellum, possibly due to autolysis and consequent RNA degradation, but the autopsy findings showed no signs of inflammation or necrosis in these tissues; and b) viral invasion of postmortem fetal tissues is not necessarily indicative of infection, as it could have happened after the fetal death, but on close histological observation, the lungs showed thickening of alveolar walls, which may be indicative of an active inflammatory process, which had been occurring on the fetus. This can be corroborated by the presence of lymphocytes in the lung parenchyma, which is consistent with response to viral infections.

In this case report, we demonstrated a clear association between maternal SARS-CoV-2 infection, mother-to-child transmission, and fetal death, probably caused by massive thromboembolic involvement of the placenta, a complication of COVID-19 in pregnancy. This case demonstrated the need to closely monitor pregnant women with COVID-19, with scheduled returns at shorter intervals for clinical, laboratory, and imaging reevaluation. The use of MRI to assess fetal–placental function and laboratory investigation of coagulation and inflammatory biomarkers can help monitor pregnant women with COVID-19 with high-risk factors to avoid unfavorable outcomes, as shown in this case.

## Data Availability Statement

The original contributions presented in the study are included in the article/Supplementary Material, further inquiries can be directed to the corresponding author/s.

## Ethics Statement

This study was reviewed and approved by the Research Ethics Committee of the Federal University of Rio de Janeiro Maternity School. Written informed consent was obtained from the patient for the publication of this case report.

## Author Contributions

AP-B, AC, and PM conceived the study and edited the manuscript in its final form. PM wrote the first draft of the manuscript. PO-S, SG, and FT-M performed and analyzed the fetal–placental magnetic resonance image. EA-P and FA performed the autopsy. EA-P analyzed the placenta. LC and FA performed the macroscopic examination of the fixed brain and selected material from the general autopsy for histological and immunohistochemical analysis. MM, MG, IG, LS, and SR performed the biomolecular tests and fluorescence *in situ* hybridization (FISH) for the detection of SARS-CoV-2 in the placenta and fetus. MO and JA contributed to data collection and logistical organization. All authors contributed to the writing of the manuscript, read, and approved its final format.

## Conflict of Interest

The authors declare that the research was conducted in the absence of any commercial or financial relationships that could be construed as a potential conflict of interest.

## Publisher's Note

All claims expressed in this article are solely those of the authors and do not necessarily represent those of their affiliated organizations, or those of the publisher, the editors and the reviewers. Any product that may be evaluated in this article, or claim that may be made by its manufacturer, is not guaranteed or endorsed by the publisher.
