# Supplementary material for: Case Report: SARS-CoV-2 Mother-to-Child Transmission and Fetal Death Associated With Severe Placental Thromboembolism
Source: Front Med (Lausanne). 2021 Aug 16;8:677001. doi: 10.3389/fmed.2021.677001 (PMC8415358; doi:10.3389/fmed.2021.677001)
Supplement: Supplementary file 1 [file Table_1.DOCX]

Supplementary Material

**IMAGING AND LABORATORY PROTOCOLS**

1. **Protocol for fetal-placental Magnetic Resonance Imaging (MRI)**

An advanced MRI protocol, as part of an ongoing trial to study COVID-19 fetal and prenatal manifestations, was performed in a 3.0 Tesla MRI scanner (Magnetom Prisma, Siemens Healthcare, Erlangen, Germany) using two 18-channels abdominal coils, after the administration of 1 mg of Flunitrazepam (Rohypnol®️, Roche, Basel, Switzerland) to the patient. Multiple sequences were performed for detailed analyses of the placenta and fetal organs in the three planes, as described below:

1. Placenta: T2-weighted Half-Fourier Acquired Single-shot Turbo spin-Echo sequence (T2W-HASTE), Time to repeat/Time to Echo (TR/TE)= 1100/132ms, Average= 1, matrix= 256 x 256, voxel resolution= 5 x 1.56 x 1.56 - in the three planes) and a sagittal T1-weighted Volumetric Interpolated Breath-hold Dixon method sequence (T1W-VIBE DIXON), TR/TE= 520/1.9ms, Average= 1, matrix= 256 x 256, voxel resolution= 2.5 x 1.29 x 1.29;
2. Fetus Head: T2W-HASTE coronal of three planes, T2-True Fast Imaging With Steady-state Precession sequence (T2W-TRUFI) of three planes, T1W DIXON-VIBE sagittal, TR/TE= 4.4/1.3ms, Average= 1, matrix= 288 x 288, voxel resolution= 2.5 x 1.27 x 1.27, T1-weighted Gradient spin echo (T1W-GRE) axial, TR/TE= 149/4ms, Average= 1, matrix= 256 x 256, voxel resolution= 4 x 1.29 x 1.29, Intravoxel Incoherent Motion diffusion weighted (IVIM DWI) 2D diffusion weighted Trace and Apparent Diffusion Coefficient (ADC) map, TR/TE= 1400/45ms, Average= 1, matrix= 102 x 128, voxel resolution= 5 x 3.125 x 3.125, b value= 0 / 100 / 1000s/mm2;
3. Body: T2w HASTE of three planes;
4. Spine: T2W TRUFI 3D.

Acquisition time around 40 minutes.

1. **Protocol for detection of SARS-CoV-2 in the placenta and fetus**

Freshly collected samples from the fetal and maternal faces of the placenta, umbilical cord, heart, lungs, liver, kidney, intestines, cerebellum, olfactory bulb, and brain cortex were properly stored in RNAlater (Thermo Fisher Scientific, USA). The specimens were thickly sliced and separately transferred to BeadBugTM prefilled tubes with 3.0 mm Zirconium homogenizer beads (Benchmark Scientific Inc, USA) and mixed energetically with the BeadBug Microtube Homogenizer D1030-E (Benchmark Scientific Inc, USA). Then, total RNA was isolated with 1 mL of TRIzol Reagent (Thermo Fisher Scientific, USA), following the manufacturer’s instructions. The SARS-CoV-2 qRT–PCR detection assays were conducted with the 2019–nCoV CDC RUO Kit 10006713 (Integrated DNA Technologies [IDT], USA) with primes and probes for the detection of viral RNA (nucleocapsid N1 and N2 fragments) and the RNase P (RP) primer set for the detection of human RNase P RNA. The RT-qPCR reactions had a total volume of 20 µL, consisting of 15 µL of GoTaq® Probe 1-Step RT-qPCR System A6120 (Promega Corporation, USA) comprised of the following components: 3.1 µL ultrapure water, 10 µL GoTaq® Probe qPCR Master Mix with dUTP (2x), 0.4 µL GoScriptTM RT Mix for 1-Step RT-qPCR, 1.5 µL primer/probe sets for either N1, N2, or RP (IDT) and 5 µL of extracted RNA. Every reaction had appropriate internal negative controls (human specimen controls: neural stem cell; a pediatric post mortem kidney tissue of a non-Covid four-month-old infant, who died due to respiratory failure caused by bilateral pneumonitis; and adult nasopharyngeal swabs, all negative for respiratory viruses), no template control (nuclease free water), and positive controls (2019-nCoV_N Positive Control plasmid, IDT 10006625; Hs_RPP30 Positive Control, IDT 10006626), and positive adult nasopharyngeal swabs. The thermocycler (StepOnePlusTM Real-Time PCR System thermocycler – Thermo Fisher Scientific, USA) was set for a holding stage at 45°C for 15 min, 95°C for 2 min, followed by 45 cycles of denaturation at 95°C for 3 seconds, and annealing and extension at 55°C for 30 seconds. The mean and standard deviations of cycle threshold (Ct) were calculated from results obtained in all analyzed distinct fragments from the same specimens. The interpretation of the results was based on the Inform Diagnostics SARS-CoV-2 RT-PCR Assay (1), which considers as a positive result if either N1 or N2 Ct value is less than 40. The standard curve and data analysis was prepared as described previously (2). The mean and standard deviations of absolute quantification (number of copies/reactions) of N1 and N2, and the mean and standard deviations of crossing points (Cp) values of Hs_RPP30 were calculated from data obtained in all analyzed distinct fragments from the same postmortem tissue.

1. Inform Diagnostics, Inc. Inform Diagnostics SARS-CoV-2 RT-PCR Assay Early Use Authorization Summary. [Internet]. Available from: https://www.fda.gov/media/139572/download.
2. Gomes IC, Karmirian K, Oliveira J, Pedrosa C, Rosman FC, Chimelli L, Rehen S. SARS-CoV-2 Infection in the Central Nervous System of a 1-Year-Old Infant Submitted to Complete Autopsy. Preprints 2020, 2020090297. doi: 10.20944/preprints202009.0297.v1.
3. **Protocol for immunostaining against SARS-CoV-2 Spike protein**

Paraffin blocks from lungs, heart and brain were sectioned at 4 μm and deparaffinized, rehydrated and treated with 10 mM citrate buffer (pH 6.0) to achieve antigen retrieval for 30 minutes at 98°C. Then the sections were blocked/permeabilized with 3% bovine serum albumin/0.3% Triton X-100 for 1h, following overnight incubation at 4°C with anti-SARS-CoV-2 spike protein monoclonal antibody (SP - GTX632604 at 1:250). In the following day, the slides were washed with PBS and incubated for 45 minutes at 37°C with secondary antibody (Goat anti-Mouse Alexa Fluor 488, A-11001 at 1:400). Nuclei were counterstained with 0.5 μg/mL 4′-6- diamino-2-phenylindole (DAPI) for 10 minutes and the slides were mounted with Aqua-Poly-mount (Polysciences).

**Supplementary Figures**


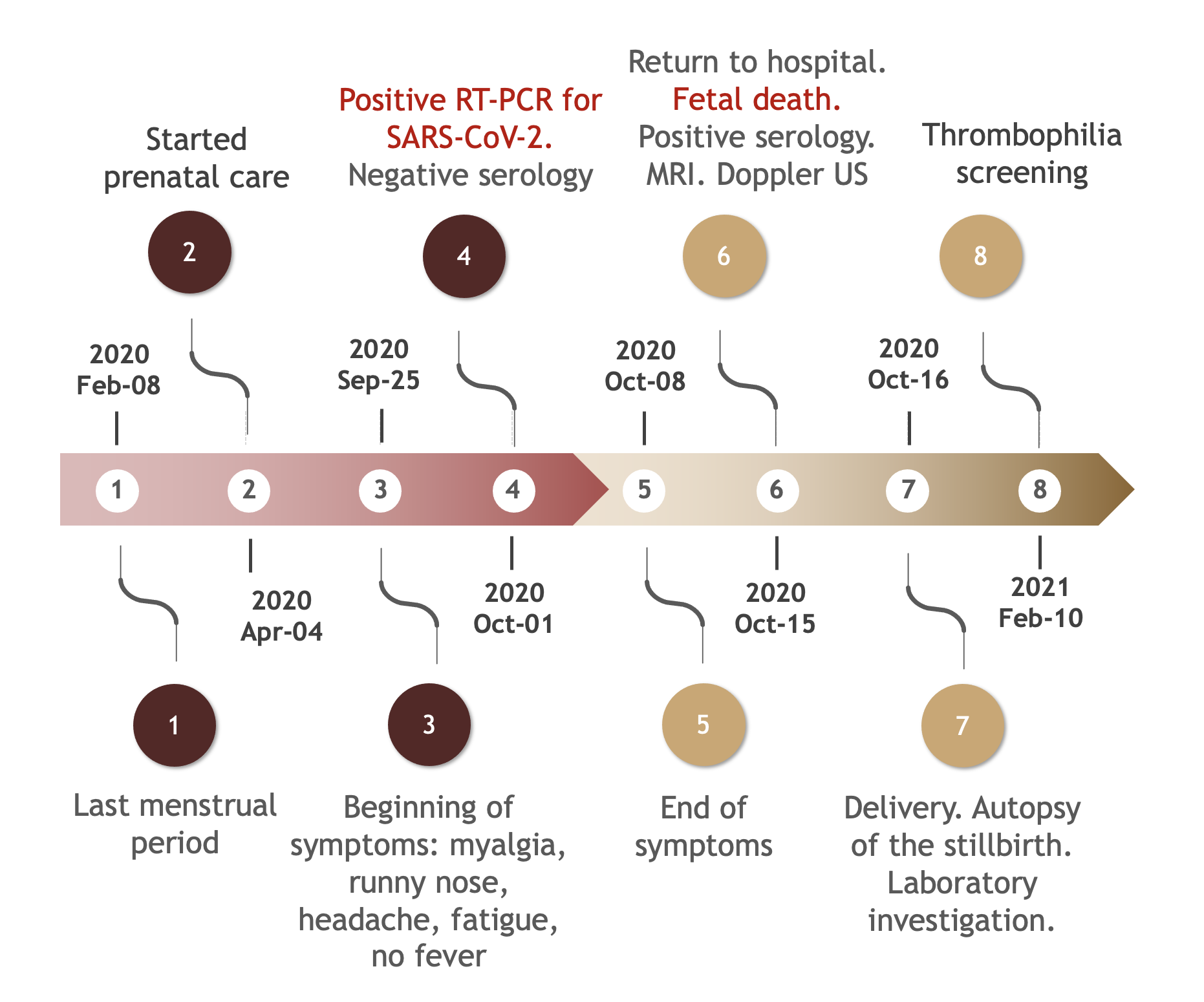


**FIGURE S1 |** Timeline of the case presentation.


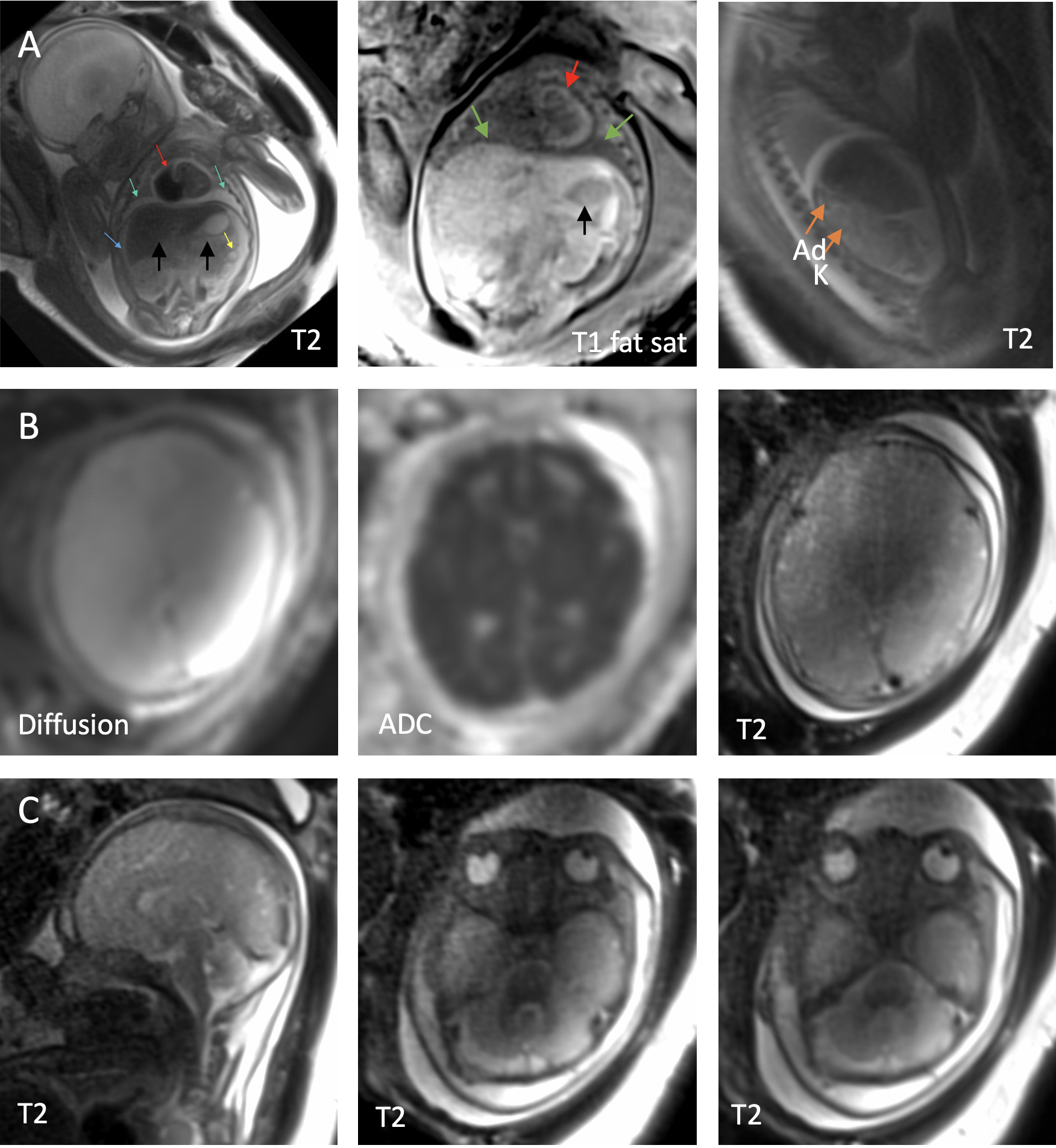


**FIGURE S2** | Fetal magnetic resonance imaging (MRI). **(A)** Liquid collection occupying 1/3 of the pleural cavity of both hemithorax (green arrows), with consequent atelectasis of both lungs, which maintains the usual morphology, but with a change in signal, characterized by a reduction in T2 and heterogeneous signal in T1 with saturation, highlighting areas of marked hypersignal, which must correspond to foci of necrosis or post-mortem autolysis. Liquid collection distending the pericardial sac (red arrows), with hypersignal in T2 and hyposignal in T1 without and with fat saturation. The cardiac muscle shows abnormal signal (hypersignal T2 and hyposignal T1 with and without fat saturation), being a sign of the presence of hemorrhage or hemorrhagic necrosis. Diffuse liquid collection throughout the peritoneal cavity, with homogeneous hypersignal in T2 and hyposignal in T1 (blue arrows). Dilatation and parietal thickening of the intestinal loops, notably jejunal and left colon (red arrows), showing content with hypersignal in T2 and hyposignal in T1, featuring a high-water content, that is, a greater amount of amniotic fluid with a minimum amount of meconium. Spleen with intermediate/hypersignal in T2 and marked hypersignal in T1 fat sat (black arrows). Liver with hyposignal in T2 and heterogeneous hypersignal in T1 fat sat (black arrows). Kidneys and adrenals with preserved signal (orange arrows). **(B)** Thickened pericranial soft tissues (subcutaneous edema). Cerebral hemispheres present loss of cortico-subcortical differentiation, associated with the erasure of the grooves between the cortical gyri, filiform lateral ventricles, and juxtaposition of the parenchyma to the inner face of the skullcap, that is, a marked reduction in the periencephalic space, denoting diffuse cerebral edema. There is an absence of delimitation of the internal and external capsules (usually detectable at this gestational age), with only a slight difference in signal highlighting both the thalamus and the peduncles of the brain. **(C)** Brainstem and cerebellum with preserved morphological and signal aspect, as well as the adjacent periencephalic space.


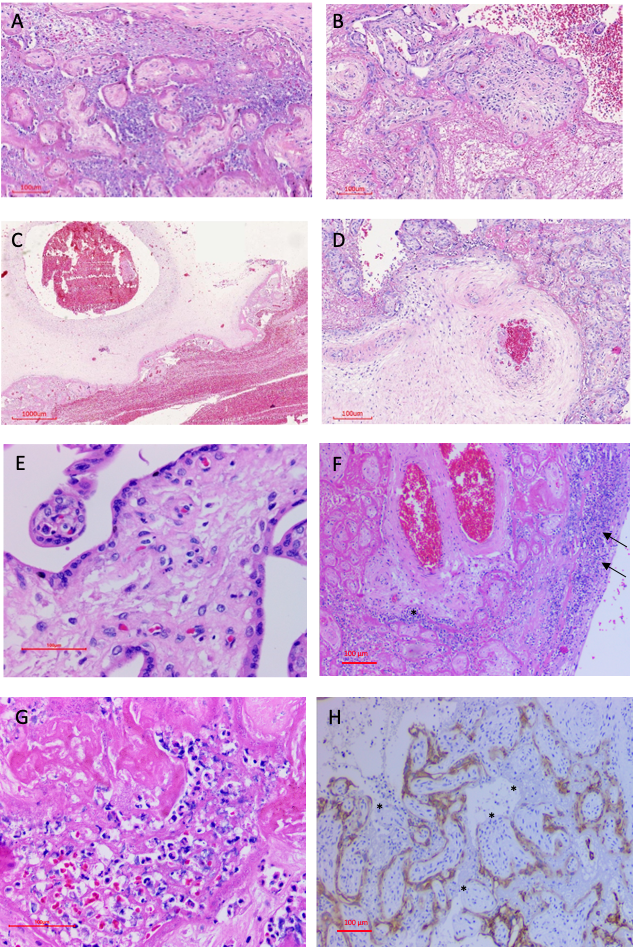


**FIGURE S3** | Histological section of the placenta.

**(A)** Intervillous space with acute intervillitis, neutrophilic exsudate and villous necrosis. **(B)** Chronic villitis and intervillous thrombosis. **(C)** Congested fetal chorionic plate vessel and subchorionic thrombosis. **(D)** Acute fetal vascular malperfusion represented by stem villous vessel with subintimal fibrin deposition and occlusive thrombus with recanalization. **(E)** Stromal-vascular kariorrhexis (arrow). **(F)** Intervillous space with acute intervillitis (*), acute deciduitis (arrow) and intervillous fibrin deposition with villous necrosis (left). **(G)** Acute intervillositis with fibrin, neutrophils and red-blood-cells in the intervillous space. **(H)** Immunohistochemistry for CK7 is positive (brown) in trophoblast cells. Some villi show fails or absence of trophoblastic layer that is an evidence of trophoblastic necrosis (*).

**
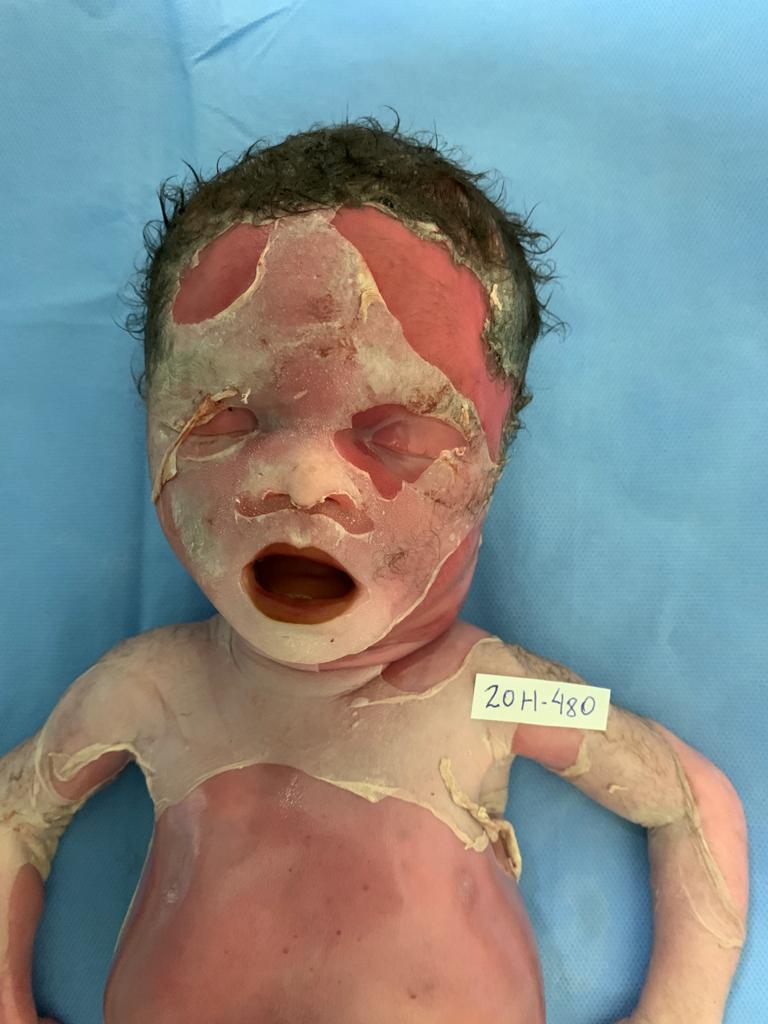
**

## FIGURE S4 | Frontal view of the stillborn with signs of maceration. The skin is detached from the surface of the body due to *post-mortem* autolysis.

**
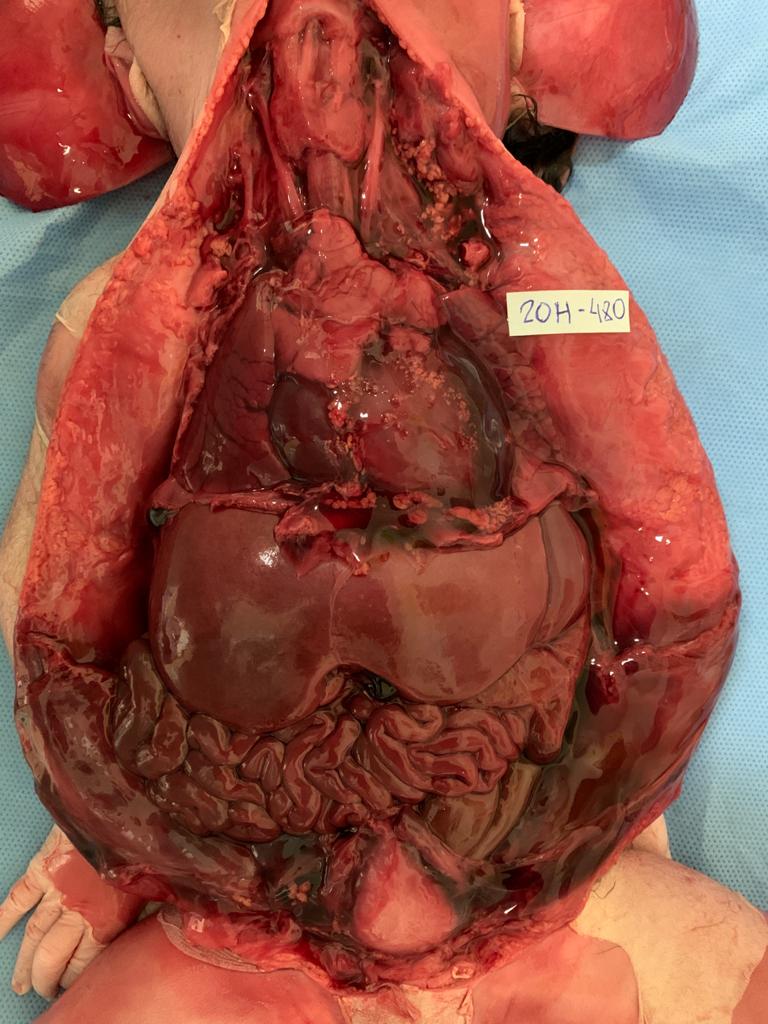
**

**FIGURE S5** | Frontal view of the thoracic and abdominal cavities. All organs are congested and soft due to *post-mortem* autolysis.


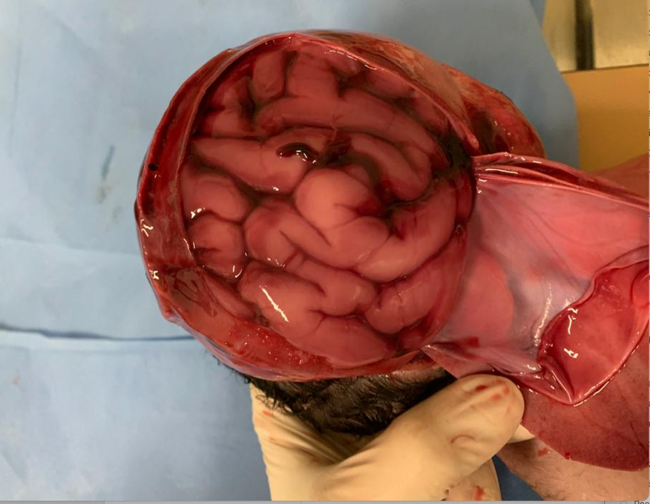


**FIGURE S6 |**External view of the cerebral surface after opening of the cranial cavity. The gyri are wide, soft and congested.

**A brief review of the most relevant literature on placental involvement and possible mother-to-child transmission of SARS COV 2.**

In the present case, the placental histopathological lesions demonstrated diffuse signs of inflammation and features of maternal and fetal vascular malperfusion. Most of these findings, especially chronic histiocytic intervillositis with trophoblast necrosis and widespread increase in fibrin deposition, with maternal vascular malperfusion, have already been observed in placentas of pregnant women infected by the coronavirus.

In September 2020, Hosier et al. (1) described a case of SARS-CoV-2 localized predominantly to syncytiotrophoblast cells at the maternal-fetal interface of the placenta. Histological examination of the placenta revealed a dense macrophage infiltrate but no evidence for the vasculopathy typically associated with preeclampsia, highlighting the potential for severe morbidity among pregnant women with COVID-19.

In November 2020, Schwartz & Morotti (2) described that the placenta shows prominent positivity of syncytiotrophoblast by SARS-CoV-2, fulfilling the published criteria for transplacental viral transmission as confirmed in fetal cells through identification of viral antigens by immunohistochemistry or viral nucleic acid using RNA in situ hybridization. These methodologies can precisely identify viruses within specific cell types and define anatomic compartments of the placenta and help explain this infection's physiopathology. The co-occurrence of chronic histiocytic intervillositis and trophoblast necrosis appears to be a risk factor for placental infection with SARS-CoV-2 and maternal-fetal viral transmission. It suggests a potential mechanism by which the coronavirus can breach the maternal-fetal interface.

Also in November 2020, Sharps et al. (3) wrote a review article summarizing placental morphology and histopathological lesions associated with SARS-CoV-2 infection. Twenty studies reported placental histopathology findings in third-trimester placentas following maternal SARS-CoV-2 infection. They found evidence of both fetal vascular malperfusion (35.3% of cases) and maternal vascular malperfusion (46% of cases), along with evidence of inflammation in the placentas (villitis 8.7% cases, intervillositis 5.3% of cases, chorioamnionitis 6% of cases). The placental pathologies observed in SARS-CoV-2 were consistent with findings following maternal SARS-CoV-1 infection. Of those tested, a minority of neonates (2%) and placental samples tested positive for SARS-CoV-2 infection (21%).

In December 2020, Edlow et al. (4) quantified SARS-CoV-2 viral load in maternal and neonatal biofluids, transplacental passage of anti-SARS-CoV-2 antibody, and incidence of fetoplacental infection in 127 patients. They concluded that in this cohort study, there was no evidence of placental infection or definitive mother-to-child transmission of SARS-CoV-2. Transplacental transfer of anti-SARS-CoV-2 antibodies was inefficient. Lack of viremia and reduced coexpression and colocalization of placental ACE 2 and transmembrane serine protease 2 may serve as protective mechanisms against vertical transmission.

In February 2021, Hsu et al. (5) describe the first case of placental SARS-CoV-2 despite mild COVID-19 disease and demonstrate placental vasculopathy and presence of SARS-CoV-2 virus across the placenta. In May 2021, Rosen et al. (6) published a prospective cohort study of pregnant women with a laboratory-proven SARS-COV-2 infection contracted before 26 weeks gestation. Women were followed at a single tertiary medical center by serial sonographic examinations every 4-6 weeks to assess fetal well-being, growth, placental function, anatomic evaluation, and signs of fetal infection. Amniocentesis and fetal brain magnetic resonance imaging (MRI) was performed at 30-32 weeks’ gestation and concluded that SARS-CoV-2 infection at early pregnancy was not associated with vertical transmission and resulted in favorable obstetric and neonatal outcomes.

There are few studies comparing placentas at term from pregnant women with COVID-19 with control groups. Patberg et al. (7) compared 77 placentas from pregnant women at term (asymptomatic and symptomatic patients) with COVID-19 with a control group (n=56). They observed that although all neonates born to mothers with coronavirus disease 2019 were negative for the SARS-CoV-2 by RT-PCR, the placentas from the studied group were associated with increased rates of histopathologic abnormalities, particularly fetal vascular malperfusion and villitis of unknown etiology. Nevertheless, Smithgall et al. (8) could not find definite evidence of SARS-CoV-2 in the placentas from positive women in their third trimester when compared with 25 singletons, third‐trimester placentas from SARS‐CoV‐2‐negative women by In Situ Hybridization and Immunohistochemistry, but they were more likely to show evidence of nonspecific histomorphology changes suggestive of maternal/fetal vascular malperfusion. The most significant findings were villous agglutination and subchorionic thrombi. In this study, all neonates tested negative for SARS-CoV-2, and all mothers recovered clinically.

In a small case series of five fetal death occurring between 21 and 38 weeks of gestation of mothers who had mild to moderate forms of COVID-19 and were all overweight or obese, Richtmann & Morotti (9) demonstrated that the most relevant placental histopathological findings were acute chorioamnionitis, massive deposition of fibrin, mixed intervillositis and villitis and one fetus had neutrophils inside alveolar spaces. Similar placental histopathological findings, represented by acute and chronic intervillositis and diffuse intervillous fibrin deposition with infarction, were described by Vivanti et al. (10) in a case of proven transplacental transmission of SARS-CoV-2 from a pregnant woman with respiratory symptoms during late pregnancy who delivered a male baby at 35w+5d; birth weight 2540 grams with Apgar scores of 4 and 2, at 1 and 5 minutes, and needed active resuscitation. RT-PCR was highly positive for COVID-19 in the placental tissue and both maternal and neonatal blood samples. The neonate presented with neurological manifestation on the second day of life (irritability, axial hypertonia, and opisthotonos), but he gradually recovered and was discharged from the hospital after 18 days.

**References**

1. Hosier H, Farhadian S, Morotti RA, Deshmukh U, Lu-Culligan A, Campbell KH, et al. SARS-CoV-2 infection of the placenta. J Clin Invest (2020) 130(9):4947-4953.
2. Schwartz DA, Morotti D. Placental Pathology of COVID-19 with and without Fetal and Neonatal Infection: Trophoblast Necrosis and Chronic Histiocytic Intervillositis as Risk Factors for Transplacental Transmission of SARS-CoV-2. Viruses (2020) 12:1308. doi:10.3390/v12111308.
3. Sharps MC, Hayes DJL, Lee S, Zou Z, Brady CA, Almoghrabi Y, et al. A structured review of placental morphology and histopathological lesions associated with SARS-CoV-2 infection. Placenta (2020) 101:13-29.
4. Edlow AG, LI JZ. Collier ARY, Atyeo C, James KE, Boatin AA, et al. Assessment of maternal and neonatal SARS-CoV-2 viral load, transplacental antibody transfer, and placental pathology in pregnancies during the COVID-19 pandemic. JAMA Netw Open (2020) 3(12):e2030455.
5. Hsu AL, Guan M, Johannesen E, Stephens AJ, Khaleel N, Kagan N, et al. Placental SARS-CoV-2 in a pregnant woman with mild COVID-19 disease. J Med Virol (2021) 93(2):1038-1044.
6. Rosen H, Bart Y, Zlatkin R, Ben-Sira L, Bashat DB, Amit S, et al. Fetal and perinatal outcome following first and second trimester COVID-19 infection: Evidence from a prospective cohort study. J Clin Med (2021) 10(10):2152.
7. Patberg ET, Adams T, Rekawek P, Vahanian SA, Akerman M, Hernandez A, et al. Coronavirus disease 2019 infection and placental histopathology in women delivering at term. Am J Obstet Gynecol (2021) 224(4):382.e1-382.e18.
8. Smithgall MC, Liu-Jarin X, Hamele-Bena D, Cimic A, Mourad M, Debelenko L, et al. Third-trimester placentas of severe acute respiratory syndrome coronavirus 2 (SARS-CoV-2)-positive women: histomorphology, including viral immunohistochemistry and in-situ hybridization. Histopathology (2020) 77(6):949-999.
9. Richtmann R, Torloni MR, Oyamada Otani AR, Levi JE, Crema Tobara M, de Almeida Silva C, et al. Fetal deaths in pregnancies with SARS-CoV-2 infection in Brazil: A case series. Case Rep Womens Health (2020) 27:e00243. doi.org/10.1016/j.crwh.2020.e00243.
10. Vivanti AJ, Vauloup-Fellous C, Prevot S, Zupan V, Suffee C, Do Cao J, et al. Transplacental transmission of SARS-CoV-2 infection. Nat Commun (2020) 11(1):3572.
